# Endogenous progesterone in unexplained infertility: a systematic review and meta-analysis

**DOI:** 10.1007/s10815-022-02689-5

**Published:** 2022-12-27

**Authors:** Claudia Raperport, Elpiniki Chronopoulou, Roy Homburg, Khalid Khan, Priya Bhide

**Affiliations:** 1 Women’s Health Research Unit, Wolfson Institute for Population Health, Queen Mary University of London, London, UK; 2 London North West Hospitals NHS Trust, London, UK; 3 Centre for Reproductive and Genetic Health, London, UK; 4 Hewitt Fertility Centre, Liverpool Women’s Hospital, Liverpool, UK; 5 Department of Preventative Medicine and Public Health, Faculty of Medicine, University of Granada, Granada, Spain; 6 CIBER Epidemiology and Public Health, Madrid, Spain; 7 Fertility Unit, Homerton University Hospital NHS Trust London, London, UK

**Keywords:** Endogenous progesterone, Unexplained infertility, Endometrial biopsy, Steroid hormones, Receptivity

## Abstract

**Purpose:**

To investigate the possibility that altered actions of endogenous progesterone affect receptivity and contribute to unexplained infertility (UI).

**Methods:**

Two authors electronically searched MEDLINE, CINAHL and Embase databases from inception to 6 July 2022 and hand-searched according to Cochrane methodology. We included all published primary research reporting outcomes related to endogenous progesterone in natural cycles in women with UI. Studies were assessed for risk of bias using a modified Newcastle–Ottawa Score or NHLBI Score. We pooled results where appropriate using a random-effects model. Findings were reported as odds ratios or mean differences.

**Results:**

We included 41 studies (*n* = 4023). No difference was found between the mid-luteal serum progesterone levels of women with UI compared to fertile controls (MD 0.74, − 0.31–1.79, *I*^2^ 36%). Women with UI had significantly higher rates of ‘out-of-phase’ endometrium than controls. Nine out of 10 progesterone-mediated markers of endometrial receptivity were significantly reduced in women with UI compared to fertile controls (the remaining 1 had conflicting results). Resistance in pelvic vessels was increased and perfusion of the endometrium and sub-endometrium reduced in UI compared to fertile controls in all included studies. Progesterone receptor expression and progesterone uptake were also reduced in women with unexplained infertility.

**Conclusions:**

End-organ measures of endogenous progesterone activity are reduced in women with UI compared to fertile controls. This apparently receptor-mediated reduction in response affects endometrial receptivity and is implicated as the cause of the infertility. Further research is required to confirm whether intervention could overcome this issue, offering a new option for treating unexplained infertility.

**Trial registration:**

PROSPERO registration: CRD42020141041 06/08/2020.

**Supplementary Information:**

The online version contains supplementary material available at 10.1007/s10815-022-02689-5.

## Introduction

Unexplained infertility (UI) is a diagnosis of exclusion given to all couples without a proven reproductive pathology, who fail to conceive spontaneously. It affects up to 30% of couples referred to reproductive medicine units [1–3]. It is unlikely that there is a ‘one-answer-fits-all’ explanation for unexplained infertile couples.

Fertilisation and implantation are difficult to assess and implantation problems are often assumed to be implicated in the causality of UI. Implantation failure may be related to either embryonic factors or endometrial environment, which is the focus of this review. This review investigates the possibility that some women have reduced progesterone-mediated receptivity affecting implantation.

Progesterone is mainly responsible for endometrial secretory transformation and establishment of receptivity but also affects ciliary action and muscular contraction of the fallopian tubes facilitating embryonic transport [4, 5]. The role of endogenous progesterone in human reproduction is myriad and not fully understood. Progesterone is associated with multiple molecular pathways and its actions are expressed through a complex network of downstream molecules [4]. Progesterone function is reflected in changes at the level of the endometrium, which can be assessed following endometrial biopsy and endometrial dating [5]. It also influences pelvic organ perfusion by reducing vascular resistance, which can be assessed using ultrasound [6].

The existence of a ‘luteal phase defect’ (LPD) was first described in 1949 [7] and remains a controversial issue. It describes a reduced response to progesterone causing symptoms including spotting or a short luteal phase. Many specialists refute the possibility of defective progestogenic action despite normal ovulation [8].

Defective progestogenic action could refer to reduced production or impaired downstream response, reducing the receptivity of the endometrium and subsequently the likelihood of conception.

## Methods

The review protocol was written and registered with PROSPERO prospectively: CRD42020141041. The paper was reported according to the 2009 PRISMA statement [9].

### Eligibility criteria, information sources and search strategy

Two authors (CR and EC) independently searched the following electronic databases (from inception to 1 July 2022) for all relevant published literature: MEDLINE In-Process & Other Non-Indexed Citations, Ovid platform, Embase, Ovid platform, Cumulative Index to Nursing and Allied Health Literature (CINAHL), EBSCO platform.

We used both electronic searches of bibliographic databases and hand-searching as described in the Cochrane Handbook for Systematic Reviews of Interventions [10].

The comprehensive search strategy was designed to reduce selection bias and to include variations in terms used and international spellings.

The search strategies are attached as Appendix S1. Search result reporting was conducted in accordance with PRISMA 2009 guidance prior to publication of the updated guidance [11]. An up-to-date search was performed immediately before submission for publication.

We included articles describing human participants, published as full manuscripts in the English language. Inclusion criteria were studies assessing women with unexplained infertility (all definitions included) and with no medical intervention. For comparative studies, the comparator/control groups included either fertile women or women with a different subfertility diagnosis.

There is no single accepted definition for unexplained infertility; therefore, we gathered all studies describing unexplained or idiopathic infertility which stated a clear set of diagnostic criteria.

### Study selection

Two authors (CR and EC) independently and manually screened all titles according to the inclusion criteria.

Abstracts were then screened for the same criteria and checked to ensure that full papers were accessible. The full papers were then obtained and screened thoroughly through assessment of materials and methods sections and inclusion/exclusion criteria and appropriate results. Any disagreements between the authors were settled through discussion and where necessary, the third author (PB) was consulted.

### Data extraction

Raw data including demographics, baseline clinical results and observational data were entered into a bespoke Excel spreadsheet designed to suit the heterogeneous nature of this study, with each included study requiring individual results sections.

### Main outcome measures

Outcomes were measurable evidence of the effects of endogenous progesterone (Table 1). These included mid-luteal progesterone levels (serum, peritoneal and salivary).

Ultrasound studies reported endometrial thickness and character, and measures of perfusion including uterine, ovarian and spiral artery resistance, endometrial and sub-endometrial perfusion.

Endometrial biopsy results were reported, measuring endometrial dating, steroid hormone receptors, endometrial protein PP14, α1 and β3 integrins, GP130 and progesterone inhibitory blocking factor (PIBF), ghrelin hormone, gene expression for SGK1 enzyme and SOCS1, progesterone receptor polymorphisms, monoclonal antibody D9B1, leukaemia inhibitory factor (LIF) and pinopode structures.

**Table 1:** Included outcome measures and their relationship to progesterone

Outcome measure	Function	Relationship to progesterone
Endometrial dating	In 1975, Noyes et al. [12] combined all the current knowledge to define the characteristics of the endometrium that change rapidly enough and with enough sensitivity to steroid hormones that they can help accurately illustrate the stage of the cycle. Noyes criterion is a grading system that incorporates gland mitoses, pseudo-stratification of nuclei, basal vacuolation, secretion, stromal oedema, pseudo-decidual reaction, stromal mitoses and leucocytic infiltration. The focus of change is in the glandular epithelium in the early secretory phase and then the stromal epithelium in the late secretory phase	Endometrial transformation is progesterone-mediated
Integrins	Integrins are endometrial cell adhesion molecules; the actions of which are multi-factorial	Progesterone-mediated expression of α1β1 integrin has been demonstrated Αvβ3 is regulated by both oestrogen and progesterone [13–16]
Ghrelin	Ghrelin is a hormone associated with appetite with high levels associated with obesity and low with anorexia	Both ghrelin and GHSR (ghrelin receptor) have been found in the ovary and endometrium and animal studies have proven an inhibitory effect of ghrelin on progesterone secretion in pigs [17], goats [18], and sheep [19]. Ghrelin inhibits progesterone secretion by human placental JEG3 cells but its role in human progesterone regulation pre-pregnancy is unproven. Ghrelin dysregulation in obese/anorexic patients significantly reduces progesterone secretion (p < 0.05)
D9B1	D9B1 is a monoclonal antibody associated with secretory transformation of the endometrium and is found in the gland lumen	D9B1 peaks in the early-mid-luteal phase in association with maximal progesterone production. The exact mechanism is unknown but it is assumed that production is increased in response to progesterone secretion [20]
PP14	PP14 is also known as α2-pregnancy associated protein (α2-PEG) and is an endometrial protein associated with secretory transformation	Production is progesterone-dependent [21–24] and peaks during the secretory phase [21]. It has been linked to progesterone-dependent endometrial differentiation [25]
Gp130	Gp130 is a transmembrane protein that works as a cytokine receptor and has been implicated in regulating cytokine action during the process of blastocyst implantation	Gp130 increases during the secretory phases and has been shown to be secreted in larger amounts under the influence of progesterone [26]
LIF (leukaemia inhibitory factor)	LIF is a cytokine found in the endometrium and essential for pinopode formation and implantation [27]. Mice studies have proven that LIF deficiency completely inhibits blastocyst implantation	Murine studies have suggested that incubating uterine tissue with progesterone stimulated LIF mRNA expression [28]. Progesterone has shown to be a direct potent inducer of LIF from T cells both directly and indirectly through IL-4 mediation which is also progesterone induced [29]
SOCS1	SOCS1 is a gene associated with suppressing LIF-mediated signalling [30]	The relationship between SOCS1 and progesterone has not been studied but for the purpose of this review, the SOCS1 results are included since the protein is involved in LIF regulation which is progesterone mediated and likely to be linked [30]
Pinopodes	Pinopodes are ectoplasmic structures that protrude from endometrial epithelial cells found in receptive endometrium	Pinopode formation studies have shown that pinopode formation directly correlates with progesterone exposure [31, 32]
ER-α	Oestrogen receptors are steroid hormone receptors found in the endometrium amongst many other sites	ER-α expression is known to be downregulated by progesterone [33–35]
PROGINS PR	Progesterone receptor PROGINS polymorphism which has previously been linked to endometriosis [36–38]	PR PROGINS is a polymorphic variant of the progesterone receptor which is associated with reduced response to progestin in animal studies [39]
SGK1 gene expression	Serum and glucocorticoid-regulated kinase 1 (SGK1) is an enzyme in the serine/threonine kinase family which plays a role in endometrial regulation for implantation and pregnancy maintenance [40]; although the exact mechanisms are unclear	SGK1 gene expression rises through the secretory phase, correlating with rising serum progesterone levels. When samples of endometrium were incubated with progesterone and oestrogen, the exposure to progesterone doubled SGK1 mRNA compared to samples incubated in oestrogen which showed no increase [41]
Progesterone-induced blocking factor (PIBF)	PIBF is a progesterone-induced protein that reduces maternal immunological response to trophoblastic invasion. The mechanisms of PIBF action include inhibiting activated natural killer (NK) cells, activating T helper type 2 (Th2) cells, induces the production of interleukin (IL)-3, IL-4 and IL-10 and suppresses IL-12 [42]	PIBF is produced directly in response to rising progesterone levels in the luteal phase and pregnancy [42, 43]
Pelvic organ perfusion	In studies concerning fertility, uterine, ovarian, endometrial and sub-endometrial artery blood flow have demonstrated a significant correlation with histological and hormonal (progesterone) markers of uterine receptivity [44–46]	Endometrial and sub-endometrial perfusion increases from the 4/5th day of the luteal phase in sync with rising progesterone levels [47]

### Assessment of risk of bias

Two review authors (CR and EC) independently assessed the risk of bias for each study using the Newcastle–Ottawa Scale (NOS), ‘Coding Manual for Case–Control Studies’ [48] for all controlled studies and the National Heart, Blood and Lung Institute (NHBLI) ‘Quality Assessment Tool for Observational Cohort and Cross-sectional Studies’ criteria for those without a control group [49]. Any disagreements were resolved through discussion between the authors.

The GRADE framework was applied to measure evidence quality [50]. All risk of bias and GRADE scores are detailed in Appendix S2.

### Data synthesis

The data for each outcome was compared between groups. Any results reported as median and range or mean and standard error of the mean (SEM) were converted to mean and standard deviation (SD) using an online tool (http://www.math.hkbu.edu.hk/~tongt/papers/median2mean.html) [51–53]. If studies had homogenous cohorts and measured the same outcomes using similar methods, results were pooled for meta-analysis. The pooled estimates for outcomes were presented as odds ratios (OR) for dichotomous variables and standardised mean difference (SMD) for continuous variables with 95% confidence intervals using the random-effects model and inverse variance method. Statistical significance was assumed when *p* < 0.05. Statistical heterogeneity was assessed by measuring the *I*^2^ statistic. Substantial heterogeneity was assumed when *I*^2^ was calculated to be greater than 50%. If studies demonstrated clinical and methodological heterogeneity and deemed unsuitable for meta-analysis, a narrative review was presented.

We divided the studies into subgroups according to outcome measures and analysed the data within the bespoke spreadsheet. Heterogeneity was assessed between studies reporting the same outcomes and where relevant, meta-analysis was performed using RevMan software [54, 55] and results compared.

## Results

### Study selection

The original search retrieved 526 results from which 41 studies were selected for the review. The search and selection process were documented with a PRISMA flow chart below.
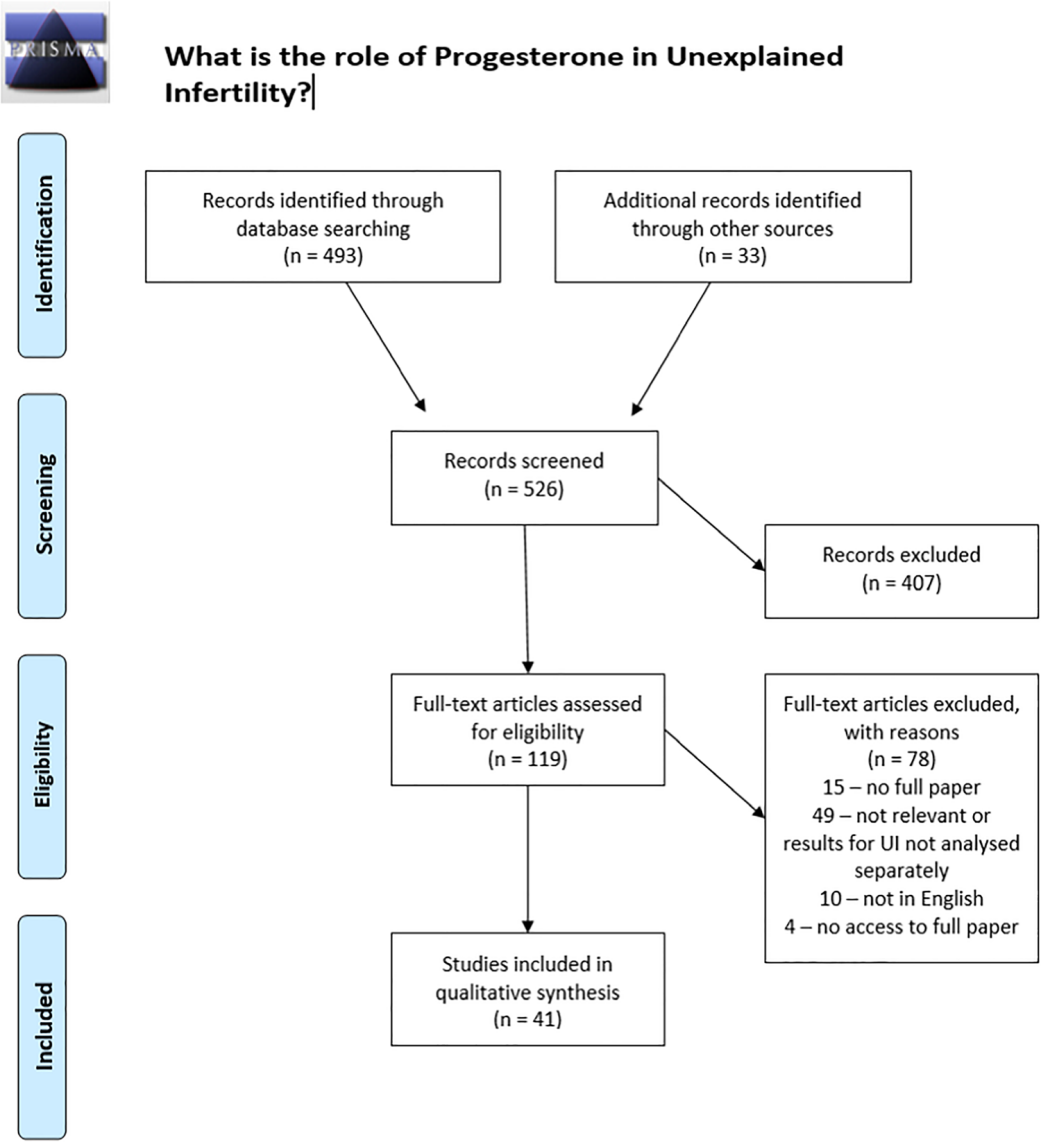


### Study characteristics

The 41 included studies were all prospective, observational studies. Five studies had no control group, 31 studies included a fertile/parous control group and five studies only had a control group with alternative infertility diagnoses. The characteristics of the included studies are detailed in Table 2 and Appendix S3.

**Table 2:** Characteristics of included studies with quality and risk of bias scores

Study ID	First author	Year	Journal	N	GRADE quality score	Risk of bias scoring system	ROB score	ROB
1	Aghajanova	2010	*Reproductive Sciences*	28	Low	NOS	4	Fair
2	Aghajanova	2009	*Fertility and Sterility*	40	Low	NOS	4	Fair
3	Ali	2021	*International Journal of Clinical Practice*	243	Low	NOS	5	Good
4	Barry-Kinsella	1994	*European Journal of Obstetrics and Gynaecology and Reproductive Biology*	54	Low	NOS	4	Fair
5	Ceydeli	2006	*European Journal of Obstetrics and Gynaecology and Reproductive Biology*	66	Low	NOS	4	Fair
6	Dhorostgoal	2017	*Int. Journal Fertil Steril*	26	Low	NOS	5	Good
7	Dixit	2018	*J Gyn Obs Hum Reprod*	45	Low	NOS	4	Fair
8	Driessen	1980	*British Journal of Obstetrics and Gynaecology*	38	Low	NOS	1	Poor
9	Du	2011	*Mol Med Reports*	300	Low	NOS	5	Good
10	El Mazny	2013	*European Journal of Obstetrics and Gynaecology and Reproductive Biology*	80	Low	NOS	5	Good
11	Feroze-Zaidi	2007	*Endocrinology*	14	Low	NOS	3	Fair
12	Gimenes	2010	*Clinical Science*	377	Low	NOS	4	Fair
13	Graham	1990	*British Medical Journal*	71	Low	NOS	4	Fair
14	Hambartsoumiam	1998	*American Journal Reproductive Immunology*	49	Low	NOS	5	Good
15	Hamilton	1990	*Fertility and Sterility*	218	Low	NOS	5	Good
16	Haxton	1987	*British Journal of Obstetrics and Gynaecology*	95	Low	NHLBI	9	Fair
17	Hirama	1995	*Fertility and Sterility*	29	Low	NHLBI	10	Fair
18	Karaoglan	2021	*Ultrastructural Pathology*	36	Low	NOS	1	Poor
19	Kilic	2007	*Acta Histochemica*	62	Low	NOS	4	Fair
20	Klentzeris	1994	*Human Reproduction*	24	Low	NHLBI	10	Fair
21	Kralickova	2006	*European Journal of Obstetrics and Gynaecology and Reproductive Biology*	251	Low	NOS	3	Fair
22	Kusuhara	1992	*Horm Res*	146	Low	NOS	2	Poor
23	Kusuhara	1992	*American Journal of Obstetrics and Gynaecology*	44	Low	NOS	0	Poor
24	Laird	1997	*Human Reproduction*	76	Low	NOS	3	Fair
25	Lessey	1995	*Fertility and Sterility*	119	Low	NOS	2	Poor
26	Li	1990	*Human Reproduction*	49	Low	NHLBI	12	Good
27	Li	1991	*Human Reproduction*	227	Low	NOS	3	Fair
28	Li	1989	*British Journal of Obstetrics and Gynaecology*	55	Low	NOS	6	Good
29	Margioula-Siarkou	2017	*Cytokine*	35	Low	NOS	5	Good
30	Maynard	1983	*The Lancet*	46	Low	NOS	3	Fair
31	Mikolajczyk	2003	*Reproductive Biology*	95	Low	NOS	3	Fair
32	Murto	2013	*Reproductive Biology and Endocrinology*	71	Low	NOS	2	Poor
33	Ordi	2002	*International Journal of Gynaecological Pathology*	100	Low	NHLBI	12	Good
34	Petousis	2018	*American Journal of Reproductive Immunology*	130	Low	NOS	4	Fair
35	Raine-Fenning	2004	*Human Reproduction*	48	Low	NOS	4	Fair
36	Sahin	2020	*European Journal of Obstetrics and Gynaecology and Reproductive Biology*	46	Low	NOS	4	Fair
37	Steck	2004	*European Journal of Obstetrics and Gynaecology and Reproductive Biology*	200	Low	NOS	4	Fair
38	Tawfeek	2012	*BMC Women's Health*	35	Low	NOS	4	Fair
39	Tsai	2000	*Journal of Assisted Reproduction and Genetics*	76	Low	NOS	5	Good
40	Uysal	2012	*Journal of the Turkish-German Gynaecol Assoc*	62	Low	NOS	6	Good
41	Zebitay	2016	*Gynaecological Endocrinology*	217	Low	NOS	6	Good

### Risk of bias of included studies

Using the NOS and NHLBI modified as described in ‘Methods’, 13/41 papers were scored ‘good’ and 22/38 ‘fair’. Only 6/41 studies were considered ‘poor’ quality. All 41 included papers were observational cohort studies and categorised as low quality using the GRADE framework.

Appendix S2 reports the risk of bias scoring systems and the scores assigned to each paper.

### Synthesis of results

Results were divided into those reporting progesterone levels, those looking at ultrasound evidence of pelvic organ perfusion and those pertaining to endometrial biopsy results.

#### Serum progesterone levels

**Fig. 1:**
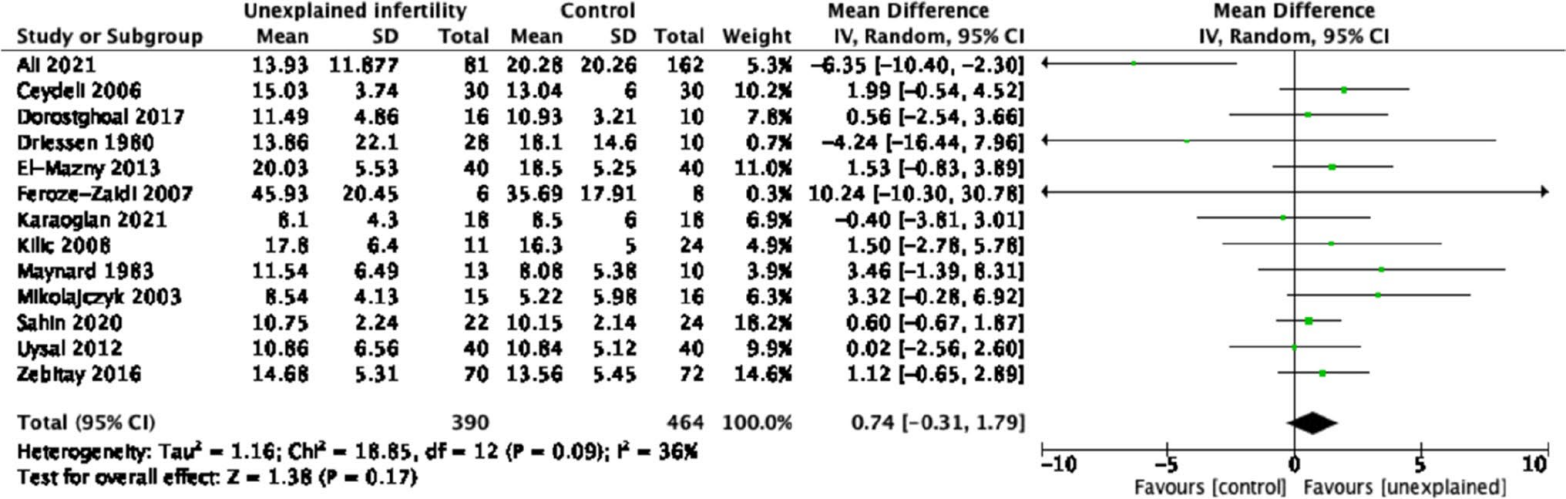
Serum progesterone levels (ng/ml) in unexplained infertility vs controls

Thirteen studies compared serum progesterone levels between women with unexplained infertility and fertile controls [41, 43, 56–68]. All 13 studies (*n* = 854) were included in the meta-analysis which reported no difference between serum progesterone levels in women with UI and controls (MD 0.74, 95% CI − 0.31–1.79, *I*^2^ 36%) (Fig. 1).

Barry-Kinsella and Kusuhara [56, 63] both compared serum progesterone levels in women with UI to those with endometriosis. Barry-Kinsella showed a significantly higher mid-luteal progesterone in the UI group compared to the endometriosis cohort (*p* < 0.03) and Kusuhara showed a non-significantly higher progesterone level in the UI group.

Hirama and Ochiai had no control group but reported a mean of 10.75 ng/ml across the entire UI cohort [69]. Murto et al. compared UI with male factor infertility and reported a mean of 10.09 ng/ml in the UI group [70].

Haxton et al. measured levels daily in women with UI and compared them against ‘normal values’. Between days LH + 1–6, 22% of women with UI had at least 3 readings below the expected values [71].

#### Peritoneal progesterone levels

A single study collected peritoneal fluid at laparoscopy in women with UI and those with mild endometriosis. They reported significantly higher levels of peritoneal progesterone in UI compared to the endometriosis group (37.11 vs 19.04 ng/ml, *p* < 0.03). No significant difference was found in serum progesterone levels (11.54 vs 10.39 ng/ml), serum estradiol (0.103 vs 0.108 ng/ml) or peritoneal estradiol levels (0.263 vs 0.242 ng/ml) [56].

#### Salivary hormone levels

Five studies (*n* = 341) measured salivary progesterone levels [72–76]. Li et al. calculated the integrated salivary progesterone concentration (the sum of the salivary progesterone levels taken daily from day LH + 1 up to and including the day before the onset of menses). This study had no control group but found significantly lower levels in women with out-of-phase endometrium (2425 pmol/l) compared to those with ‘in-phase’ endometrium (3848 pmol/l) (*p* < 0.001) [72]. Graham et al. measured salivary progesterone in UI. Five out of 23 (21.7%) women had low levels of progesterone [75].

Three studies from the same group all analysed salivary progesterone results for each day of the luteal phase. One found no significant difference overall between fertile, tubal factor infertility, male factor infertility, endometriosis and unexplained infertility groups (total *n* = 227, no raw data published) [73]. The other two studies reported that salivary progesterone levels were significantly lower in the early luteal phase in women with out-of-phase endometrial biopsy results. One showed statistically lower levels from day LH + 3–5 but from day LH + 6, no difference persisted [76]; the other found a statistical difference daily from day LH + 3–7 [74].

Of note, one study [74] identified a threshold of 300 pmol/l salivary progesterone below which endometrial dating according to Noyes criteria [12] barely changed but above which, dating stage advanced rapidly as progesterone levels increased (*p* < 0.001).

#### Steroid hormone receptor expression

Steroid hormone receptors have been studied in four papers (*n* = 220). Maynard reported a significantly higher incidence of low progesterone uptake in the UI group compared to fertile controls and those with other known infertility diagnoses in the mid-proliferative to late secretory phases (0% UI vs 43% known infertility diagnosis vs 60% fertile, *p* < 0.005) and more specifically in the late proliferative to mid-secretory phases (0% UI vs 33% known infertility diagnosis vs 50% fertile, *p* < 0.02) [64].

Two studies measuring progesterone receptor (PR) expression in the endometrium both reported significantly reduced results in UI compared to fertile controls.

Dixit et al. [77] showed a significantly lower percentage of PR staining in UI compared with controls (epithelial: 10 ± 6.26% vs 81.18 ± 33.7%, *p* < 0.001) (stromal: 54 ± 8.2% vs 78.63 ± 17.47%, *p* < 0.001). There was no significant difference in the ER in the epithelial cells, but the stromal cells also showed a significantly lower ER staining in unexplained infertile cases (40 ± 15.11% vs 75 ± 15.49%, *p* < 0.001).

Petousis et al. [78] reported epithelial endometrial expression both regarding total PR (A + B) and PR‐B receptors alone in luminal and glandular epithelium. Total PR *h*‐score in luminal epithelial cells was 106.4 ± 14.7 for cases with UI vs 219.7 ± 15.8 for controls (*p* < 0.001). Total PR *h*‐score in glandular epithelial cells was 109.7 ± 13.9 for cases with unexplained infertility vs 220.1 ± 17.2 for controls (*p* < 0.001). PR‐B *h*‐scores were 44.3 ± 13.2 (luminal) and 48.5 ± 14.2 (glandular) for women with unexplained infertility vs 164.4 ± 15.4 and 160.7 ± 12.8 for controls (*p* = 0.001 and *p* = 0.002).

Hirama and Ochiai compared nuclear and cytosol PR with serum progesterone and endometrial dating results in women with UI (*n* = 8). They reported reduced nuclear PR expression in women with low progesterone and in-phase endometrium compared with normal progesterone and out-of-phase endometrium. When comparing all in-phase and out-of-phase endometrium results regardless of serum progesterone, there was no difference between the groups. Cytosol progesterone receptors were not significantly different between the groups [69].

#### Endometrial dating

Thirteen studies reported endometrial dating. Twelve studies used Noyes criteria [12] and 1 used their own classification [72]. Ten out of 13 studies reported 20–60% of the unexplained infertile participants to have an ‘out-of-phase’ endometrium on biopsy [69, 72–74, 76, 78–82]. The remaining 3 studies reported 0% out-of-phase results in the UI group [64, 66, 83].

Six of the 12 studies also reported endometrial dating of a control group. Of these, 4 studies reported 0–4% of the fertile controls to have ‘out-of-phase’ endometrium [64, 66, 73, 83]. Petousis et al. and Margioula-Siarkou et al. both showed 20% of the fertile control group to have out-of-phase endometrium [78, 82]. Petousis et al. also reported significantly higher ‘out-of-phase’ results in endometriosis and tubal factor groups as did Li et al. [73] who reported 29% ‘out-of-phase’ samples from women with endometriosis.

**Fig. 2:**
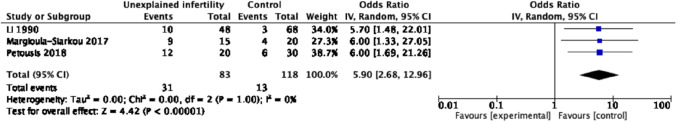
Meta-analysis of endometrial dating results

One study performed repeat biopsy in the late luteal phase and demonstrated that 24/25 ‘out-of-phase’ mid-luteal biopsies were back ‘in-phase’ a few days later [81]. Of the 6 studies with control groups, 3 reported 0% out-of-phase results excluding them from meta-analysis. The remaining 3 studies were included with an odds ratio of 5.90 (2.68–12.96), *Z* value 4.42 (*p* < 0.00001), and *I*^2^ 0% (Fig. 2).

#### Markers of receptivity

Sixteen papers (*n* = 1267) reporting on ten markers of endometrial receptivity were included in the review.

Included markers of receptivity were as follows: integrins α1 and β3 [60, 62, 80], D9B1 [75], PP14 [76], PIBF [43], SOCS1 [30], LIF [30, 65, 82, 84–89], ghrelin and its receptor GSHR [83], GP130 [30, 86] and pinopode formation [30].

##### Integrins

Kilic et al. investigated the presence of α1 integrin in the secretory phase and found significantly lower histochemical scoring (HSCORE) and semi-quantitative amounts in the stroma and glandular epithelium of the untreated unexplained infertile group when compared with fertile controls (glandular epithelium HSCORE *p* < 0.004, semi-quantitative *p* < 0.025, stromal HSCORE *p* < 0.001, semi-quantitative *p* < 0.025) [62].

One study measuring β3 integrin in the luminal epithelium showed a significantly lower HSCORE for immunostaining of 0.65 ± 0.84 for unexplained infertility compared with 2.6 ± 0.79 in the fertile group (*p* < 0.004) and 2.2 ± 0.56 for the tubal factor group (*p* < 0.001). A second study showed no significant difference in β3 expression in either the luminal, glandular or stromal epithelium [60].

##### PIBF

One study showed that the mean serum PIBF level was 6.92 ± 3.41 ng/ml in the UI group compared with 12.10 ± 10.47 ng/ml in the fertile group (*p* = 0.02) [43].

##### GP130

Gp130 immunostaining was measured by Aghajanova et al. [30] who found that fertile women have the highest intensity immunostaining in the mid-secretory phase, in both the luminal and glandular epithelium. One hundred percent fertile women showed moderate to strong immunostaining compared to 14% of infertile women in the mid-secretory phase. In the glandular epithelium, all infertile women had low immunostaining compared to 81% of fertile women showing moderate-high results.

Tawfeek et al. showed very varied results with regard after RT-PCR analysis of the two gp130 splice variants; 70% of fertile women showed very low expression of splice variant 1 and 76% infertile women showed faint expression of splice variant 1 with 30% and 24% respectively showing no expression at all. However, this same study showed significantly higher gp130 in uterine flushings from fertile women compared to unexplained infertile women (182 ± 77 vs 51.5 ± 27.5 pg/ml, *p* < 0.001) [86].

##### LIF

Two studies assessed LIF gene mutations; one found no significant difference in the number of mutations between UI and control groups [89]. The second study found 4 mutations in a cohort of 57 women with UI and 0 in the control group which was significant (*p* < 0.05) [88].

Three studies measured LIF in uterine flushings. All 3 found higher levels in the samples from fertile women compared to those with UI. One study demonstrated 57% LIF presences in the fertile group compared to 0% in the UI group on day LH + 10 [87]. Two other studies measured quantitative amounts and found lower levels in the UI group (1.57 vs 26.46 pg/ml, *p* < 0.01 and 3.9 ± 7.5 vs 48.8 ± 28.9 pg/ml, *p* < 0.001 [86]).

LIF was also measured in endometrial biopsy samples in 4 studies and all found lower levels in samples from women with UI compared to fertile controls.

Tsai et al. gave numerical scores to categories of intensity with 0 = no immunostaining and 4 = intense staining. They found a significantly higher LIF score in the control group compared with the unexplained infertile group across the luminal, glandular and stromal epithelium (*p* < 0.05) [84].

Hambartsoumian compared results at different menstrual stages as well as between groups and found a significant 2.2-fold rise in LIF from proliferative to secretory phase endometrium within the fertile population (*p* < 0.05) which was not evident in the UI group. The UI group consisted of women with unexplained infertility, some of which had attempted embryo transfer > 5 times as part of IVF treatment. This group was defined as having multiple implantation failures. In the mid-luteal phase, LIF levels were 2.2 × higher in the fertile group compared with the UI without recurrent implantation failures (*p* < 0.05) and 3.5 × higher (*p* < 0.01) than in the group with UI and multiple implantation failures [85].

One study (Tawfeek et al.) commented on the presence but not the intensity of LIF immunostaining and found 100% positive in the samples from fertile controls but only 12% positive staining in the infertile group which was statistically significant (*p* < 0.001) [86].

The fourth study demonstrated that all fertile samples showed intense LIF staining in both the glandular and luminal epithelium whereas the infertile samples showed only moderate-strong staining in 4/14 luminal and 5/14 glandular epithelial samples. Due to the small sample size, these findings were statistically insignificant [30].

##### SOCS1

There was a tendency for redistribution of apical SOCS1 mRNA from the luminal epithelium in fertile women to the glandular epithelium of infertile women. No fertile women showed almost any cytoplasmic staining in the glandular or luminal epithelium whereas 13/14 infertile women showed moderate-strong glandular immunostaining [30].

##### Pinopode formation

Eighty-eight percent of fertile women had pinopodes present compared to 57% of infertile women (Aghajanova et al.). It was noted that pinopode formation was positively correlated with LIF in the luminal epithelium (*p* = 0.01) [30].

##### D9B1

D9B1 secretory levels rose slower and to lower levels in the UI group compared to fertile controls (*p* < 0.025). The movement from intracellular to luminal levels was also decreased in the UI group suggesting defective or delayed secretion (luminal levels decreased in UI, *p* < 0.005, intracellular remaining levels higher in UI, *p* < 0.005) [75].

##### PP14

One study measured PP14 (also known asα2-PEG) in women with unexplained infertility without a true control group. They found that 8/24 women had out-of-phase endometrium [76] and these women demonstrated a significantly reduced overall rise in PP14 (170% compared to 320% in women with in-phase endometrium).

##### Ghrelin

One study reported that compared with fertile controls, women with UI showed significantly lower staining for ghrelin (*p* < 0.037) and growth hormone secretagogue receptor (GHSR—also known as ghrelin receptor) (*p* < 0.045) in the luminal epithelium. GHSR expression alone was also significantly lower in the glandular and stromal epithelium (*p* < 0.029 and 0.009) [83].

##### Genetic expression

Feroze-Zaidi et al. identified a gene expressed significantly differently between fertile women and women with UI. SGK1 was significantly upregulated in women with UI compared with fertile women (*p* < 0.05). This gene was expressed higher in secretory endometrium compared to proliferative endometrium (non-significant *p* > 0.05) correlating with rising serum progesterone levels [41].

Du et al. reported Erα polymorphisms in UI vs fertile controls [90]. Significant differences in 4 different Erα allele frequencies were found between the two groups (*p* < 0.001). Gimenes et al. found no difference in the incidence of PR PROGINS polymorphisms between fertile, UI or endometriosis groups [91].

#### Ultrasound study results

##### Endometrial thickness

**Fig. 3:**
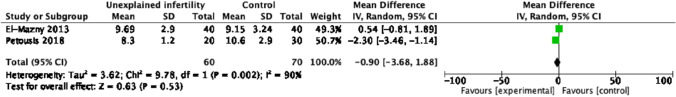
Meta-analysis of endometrial thickness results

Three studies measured endometrial thickness (ET) [47, 57, 78]. Two studies [47, 57] found no significant difference between women with UI and fertile controls in contrast to a third [78] who reported a significantly decreased ET in women with UI compared to fertile controls (8.3 ± 1.2 mm for patients with unexplained infertility vs 10.6 ± 2.9 mm, *p* < 0.03). One study [47] did not publish standard deviations and therefore could not be included in the meta-analysis. Meta-analysis reported no significant difference with a mean difference of 0.9 mm (95% CI − 3.68, 1.88, *I*^2^ 90%) (Fig. 3).

##### Pelvic organ perfusion

Four studies were included that measured pelvic organ perfusion with Doppler ultrasound studies [47, 57–59]. Three studies (*n* = 359) were included in the meta-analysis [57–59].

Three studies reported vascular perfusion (resistance index and pulsatility index) of the ovarian [59], uterine [57, 59] and spiral arteries [58, 59] using colour Doppler and analysis of waveforms of flow velocity [92].

Ovarian artery perfusion was measured in one study [59] which reported significantly increased resistance in women with UI compared to fertile controls (PI 1.06 ± 0.18 vs 0.96 ± 0.18, *p* = 0.001, ovarian artery RI 0.62 ± 0.06 vs 0.58 ± 0.06, *p* = 0.001).

Uterine artery perfusion was measured in two studies with the following results reported:

(Uterine artery PI 2.56 ± 0.68 vs 1.64 ± 0.37 *p* = 0.001 [59] and 2.12 ± 0.49 vs 1.81 ± 0.42 *p* = 0.003 [57]).

(Uterine artery RI 0.86 ± 0.05 vs 0.76 ± 0.06 *p* = 0.001 [59] and 0.89 ± 0.23 vs 0.76 ± 0.19 *p* = 0.007 [57]).

**Fig. 4:**
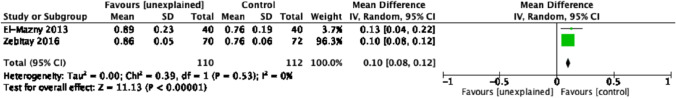
Meta-analysis of studies of uterine artery RI

**Fig. 5:**
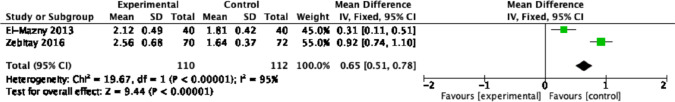
Meta-analysis of studies of uterine artery PI

Meta-analysis of these results showed a significant difference with the standardised mean difference (SMD) for uterine artery RI of 1.21 (0.05–2.37), *I*^2^ 93% and uterine artery PI SMD of 0.65 (0.51–0.78), *I*^2^ 95% (Figs. 4 and 5).

Spiral artery resistance was also measured in two studies which showed increased resistance in UI vs control:

(PI 0.80 ± 0.16 vs 0.65 ± 0.18 *p* = 0.004 [58] and 0.91 ± 0.06 vs 0.84 ± 0.13 *p* = 0.001 [59]).

(RI 0.54 ± 0.07 vs 0.48 ± 0.08 *p* = 0.009 [58] and 0.57 ± 0.03 vs 0.52 ± 0.04 *p* = 0.001 [59]).

**Fig. 6:**
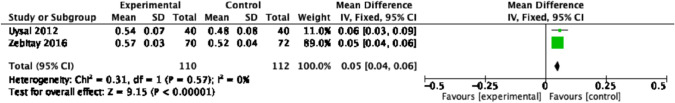
Meta-analysis of studies of spiral artery RI

**Fig. 7:**
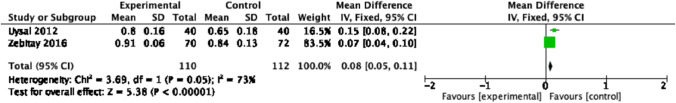
Meta-analysis of studies of spiral artery PI

Meta-analysis of the results for spiral artery resistance showed the following significant results: spiral artery RI MD 0.05 (0.04–0.06, *I*^2^ 0%) and spiral artery PI MD 0.08 (0.05–0.11, *I*^2^ 73%) (Figs. 6 and 7).

Two studies reported endometrial and sub-endometrial perfusion based on vascularisation index (VI), flow index (FI) and vascularisation flow index (VFI). Both reported reduced perfusion in UI compared with fertile controls [47, 57]. El-Mazny reported endometrial perfusion as follows: VI: UI 0.53 ± 0.18, control 0.63 ± 0.22 (*p* = 0.029); FI: UI 25.24 ± 8.57, control 29.55 ± 8.98 (*p* = 0.031); VFI: UI 0.25 ± 0.06, control 0.31 ± 0.09 (*p* = 0.001). Sub-endometrial perfusion was similarly reduced in women with UI: VI: UI 1.97 ± 0.59, control 2.27 ± 0.64 (*p* = 0.032); FI: UI 31.18 ± 10.23, control 36.70 ± 13.19 (*p* = 0.040); VFI: UI 0.83 ± 0.27, control 1.02 ± 0.31 (*p* = 0.005) [57].

Raine-Fenning et al. did not report raw data (hence, no meta-analysis was possible) but found significant reduction in endometrial VI (*p* < 0.001) and FI (*p* < 0.05) and sub-endometrial VI (*p* < 0.001) and VFI (*p* < 0.01). The VFI of the endometrium and FI of the sub-endometrium were reduced but did not reach significance in this study (*p* < 0.058, *p* < 0.088) [47].

##### Luteal cyst formation

One study reported 23.4% of women with UI had luteal cysts detected compared to 0% of fertile controls and of these, 51.2% showed some shrinkage in size after the LH surge compared to 48.8% which did not. Progesterone indices (the sum of daily levels from days LH + 2–6 and reported as a percentage of the mean levels in the control groups from the same days) in the participants with non-shrinking cysts were significantly lower than in fertile controls and in the participants with luteal cysts which had shrunk (median P index 53.5% compared with 100% and 84%) (*p* < 0.001) [93].

## Discussion

### Main findings

Despite no significant difference between mid-luteal serum progesterone levels, of 19 results measuring 10 different endometrial biomarkers, 18 reported significantly reduced levels in UI compared to fertile controls. One result for B3 integrin reported no difference giving heterogeneous results for this biomarker as the other study measuring this showed a reduction in UI.

Other downstream markers of progesterone activity including pelvic vascular flow and endometrial and sub-endometrial perfusion were also significantly reduced in women with UI as compared to controls. Receptor activity was reduced in women with unexplained infertility implicating an underlying downregulation or under-expression which leads to a reduction in endometrial receptivity which could explain the infertility.

### Interpretation of results

Either a reduction in progesterone receptor expression or a downregulation of these receptors appears to be causing a delayed or reduced receptivity response which could feasibly cause infertility for this group of women.

Endometrial dating has been demonstrated to be inadequate alone as a test to determine the cause of implantation failure [94–96]. The exact relationship between altered levels of various progesterone-mediated biomarkers and the development of the endometrium for example PP14 (levels of which are reduced in delayed endometrial development) is unclear. Similarly, although conception rates do increase with increasing endometrial thickness, alone it is not a useful marker of fertility [97] especially within medicated cycles and conception and live births can occur despite thickness below 5 mm [98].

The findings of this study suggest that further research regarding the development and receptivity of the endometrium including the various biomarkers included and pelvic organ perfusion is necessary to determine the overall difference in progesterone response in women with UI compared with fertile controls. Hopefully further research can elucidate the relationship between endometrial dating and thickness and progesterone response and receptor regulation.

In clinical practice, it is widely accepted that a single progesterone level of 30 nmol/l (9.43 ng/ml) on day 7 post-ovulation (usually referred to as day 21 assuming a regular 28-day cycle) indicates successful ovulation and luteal function.

The timing of adequate progesterone exposure may however be important, as the early luteal phase is deemed crucial for endometrial development. One study showed that low salivary progesterone levels in the early luteal phase (between day LH + 3–7) correlated with increased likelihood of delayed endometrial development [74]. It has been demonstrated that the endometrium may appear ‘out-of-phase’ in the early-mid-luteal phase and then be back ‘in-phase’ within a few days [81]. Kusuhara [79] also confirmed that within their cohort with ‘out-of-phase’ endometrium, serum progesterone levels were significantly lower than controls in the early luteal phase (days LH + 2–7) but no difference was found by the late luteal phase. One study with no control group found 22% women with UI had at least 3 days with lower than expected progesterone levels in the early luteal phase (days LH + 1–6) [71]. This compliments the findings of Cooke in 1977 [99] that suggested that infertile women had lower serum progesterone levels than fertile controls in the first but not the second half of the luteal phase. A single mid-luteal serum progesterone level is therefore of limited utility.

It is unknown whether progesterone-related expression of the various endometrial molecules and structural changes are a response to a threshold quantity or length of exposure. This concept is interesting, especially when considering that serum progesterone is mostly tested once in the mid-luteal phase which may be too late if the crucial progesterone-related development occurs before this.

Li et al. [100] documented thresholds of serum progesterone above which morphological change in the endometrium occurred—changes in the glandular vacuoles occurred above 200 pmol/l and gland lumen growth and secretion above 300 pmol/l. The threshold of 300 pmol/l was reported to be crucial for change in speed of morphometric development. It is important to note that since Noyes published his endometrial dating criteria [12], the accuracy of this has been accepted to be lower than originally believed and therefore results can be interpreted within 2 days of the expected luteal phase day rather than precise to the exact day [101]. This still allows interpretation of results to be expressed as at the expected stage (early/mid/late luteal) or delayed.

An underlying deficiency or delay in progesterone secretion in the early luteal phase could delay receptor upregulation or response in some women. The receptors may require a threshold level of progesterone at the correct time to produce the required downstream response to optimise receptivity. Timing seems to be key. If peak receptivity is delayed even by a few days, this will negatively impact the likelihood of natural conception. The window of implantation is narrow and it is possible that although progesterone secretion or receptor expression do reach threshold levels, this may be too late to achieve successful trophoblastic invasion.

### Comparison with existing literature

To our knowledge, no review has ever been published combining the different actions of progesterone in women with unexplained infertility. Since only observational studies exist, this review is important as combining the results of 41 studies with hugely varied outcome measures highlights a clear difference in physiology between fertile and infertile women not previously seen that needs further research both to clarify and also possibly to treat and reverse.

### Strengths and limitations

The search strategy was comprehensive and performed by two authors independently. Efforts were made to access the older papers that were not available in digital form. The protocol for the review was prospectively registered on PROSPERO.

One of the challenges of clinical research in the arena of subfertility is that, at the point where patients present to clinicians, they are eager to start treatment. This therefore limits the possibility of prospective studies on unmedicated ‘natural cycles’ and leads to the design and publication of small-scale, observational studies which make up the vast majority of those included in this review.

One of the criticisms of early studies involving endometrial biopsy is the lack of solid methodology regarding timings. Many studies guess a ‘late-luteal’ phase for biopsy or ‘mid-luteal’ phase for serum progesterone without accurately timing the LH surge leading to heterogeneous results [72, 102].

Publication bias could lead to misrepresentation of results if similar studies have found negative findings that were not published. Significant heterogeneity was observed in the definition of unexplained infertility. If population characteristics vary between studies, even significant findings may be diluted.

There is no single universally accepted definition of UI. Heterogeneity between the inclusion criteria for UI groups across the studies is a limitation of this and all work regarding UI.

It is well understood that progesterone in the luteal phase is released in a pulsatile fashion [103] and therefore the widespread clinical practice of measuring serum progesterone once is of limited value. Filicori’s work showed that in the mid-late luteal phase, plasma progesterone concentrations fluctuated within 24 h from lows of 2.3 to peaks of 40.1 ng/ml, often varying wildly within the space of just minutes [103].

The studies included vary drastically in terms of size with number of participants ranging from 14 to 377 and many of the included studies are over 20 years old.

The quality according to GRADE was low for all included studies due to their observational nature. Observational studies can be affected by selection bias which further decreases their utility.

## Conclusions and implications

The only test routinely performed as a measure of progesterone or progestogenic action is a mid-luteal serum progesterone. When comparing the utility of serum progesterone levels and endometrial biopsy, the end-organ (endometrium) seems more important than the hormones acting on it [74]. It is likely that any altered actions of progesterone are missed in the majority of women investigated for UI without offering an endometrial biopsy for markers of progestogenic activity and Doppler studies of the pelvic vasculature.

The findings described in this review offer a strong argument for further in-depth research into the relationship between the actions of endogenous progesterone and unexplained infertility.

Priorities for research should lie with improving the understanding of patterns of progesterone secretion throughout the luteal phase and simultaneous response in the receptors and downstream receptivity markers and pelvic perfusion.

If a relationship is proven between receptor response, progesterone secretion patterns and levels and endometrial receptivity, then this could have implications for clinical practice, especially in the use of exogenous progesterone luteal support. It would be interesting to assess whether the reduction in receptivity suggested by this study remains a concern in stimulated ART cycles or whether exogenous hormones overcome any natural deficiencies.

Literature suggests that in superovulation cycles stimulated with gonadotropins, exogenous luteal support does improve success rates [104–106]. There is minimal literature specifically looking at the effect of luteal support for UI in natural cycles or OI/IUI. Whether increasing serum levels with exogenous progesterone could upregulate receptors and hasten receptivity response remains to be proven.

## Supplementary Information

Below is the link to the electronic supplementary material.

Supplementary file 1: Prisma Checklist (DOCX 30 KB)

Supplementary file 2: Search Strategy (DOCX 21 KB)

Supplementary file 3: ROB and GRADE scoring (DOCX 270 KB)

Supplementary file 4: List of included studies (DOCX 28 KB)

Supplementary file 5: Prisma Flowchart (DOC 58 KB)
