# Correction to: Endogenous progesterone in unexplained infertility: a systematic review and meta‑analysis

**DOI:** 10.1007/s10815-023-02718-x

**Published:** 2023-01-18

**Authors:** Claudia Raperport, Elpiniki Chronopoulou, Roy Homburg, Khalid Khan, Priya Bhide

**Affiliations:** 1 Women‘s Health Research Unit, Wolfson Institute for Population Health, Queen Mary University of London, London, UK; 2 London North West Hospitals NHS Trust, London, UK; 3 Centre for Reproductive and Genetic Health, London, UK; 4 Hewitt Fertility Centre, Liverpool Women’s Hospital, Liverpool, UK; 5 Department of Preventative Medicine and Public Health, Faculty of Medicine, University of Granada, Granada, Spain; 6 CIBER Epidemiology and Public Health, Madrid, Spain; 7 Fertility Unit, Homerton University Hospital NHS Trust London, London, UK

**Correction to: Journal of Assisted Reproduction and Genetics**

10.1007/s10815-022-02689-5

In this article the affiliation details for author Claudia Raperport should have been “Women’s Health Research Unit, Wolfson Institute for Population Health, Queen Mary University of London.”

The original article has been corrected.

