# Supplementary material for: Endogenous progesterone in unexplained infertility: a systematic review and meta-analysis
Source: J Assist Reprod Genet. 2022 Dec 27;40(3):509–24. doi: 10.1007/s10815-022-02689-5 (PMC10033797; doi:10.1007/s10815-022-02689-5)
Supplement: Supplementary file 2 — Search Strategy (DOCX 21 KB) [file 10815_2022_2689_MOESM2_ESM.docx]

**The Role of Progesterone in Unexplained Infertility: A systematic review**

**Search strategy:** No intervention or comparisons were included in the search.

**The same strategy was used for Medline, EMBASE and CINAHL**

| P (Patient/Population) | O (Outcome) | O (Outcome) cont. |  |
| --- | --- | --- | --- |
| Unexplained Infertility | Embryo Implantation (MeSH) | Histology |  |
| Unexplained Subfertility | Receptivity | Receptor |  |
| Idiopathic Infertility | Clinical pregnancy | Endometrial thickness |  |
| Idiopathic Subfertility | Live birth rate | Progestogen |  |
| Infertility, female (MeSH) | Miscarriage | Perfusion |  |
|  | Progesterone (MeSH) | Integrin |  |
|  | Luteal Phase (MeSH) | ER |  |
|  | Luteal phase defect | Glycodelin |  |
|  | Luteal support | GdA |  |
|  | Endometrium (MeSH) | Cytology |  |

**Search terms used:**

Unexplained infertility OR unexplained subfertility OR idiopathic infertility OR idiopathic subfertility OR infertility, female

AND Progesterone AND

Luteal phase OR luteal support OR luteal phase defect OR endometrium OR receptivity OR embryo implantation OR progestogen OR endometrial thickness OR receptor OR histology OR cytology OR miscarriage OR clinical pregnancy OR live birth rate OR GdA OR glycodelin OR ER OR Integrin OR perfusion
