# Supplementary material for: Endogenous progesterone in unexplained infertility: a systematic review and meta-analysis
Source: J Assist Reprod Genet. 2022 Dec 27;40(3):509–24. doi: 10.1007/s10815-022-02689-5 (PMC10033797; doi:10.1007/s10815-022-02689-5)
Supplement: Supplementary file 3 — ROB and GRADE scoring (DOCX 270 KB) [file 10815_2022_2689_MOESM3_ESM.docx]

**Supplementary Materials Appendix S3**

Newcastle-Ottawa Scoring (NOS) (for trials with a control group)

All controlled studies were assessed for risk of bias using the NOS score detailed below. Comparability factors chosen were a) reproductive pathology and b) age.

This gave a maximum possible score of 6. We categorised the studies as:

‘Good’ to scores 5 and 6 out of 6

‘Fair’ to score 3 and 4 out of 6

‘Poor’ to score 0,1 and 2 out of 6


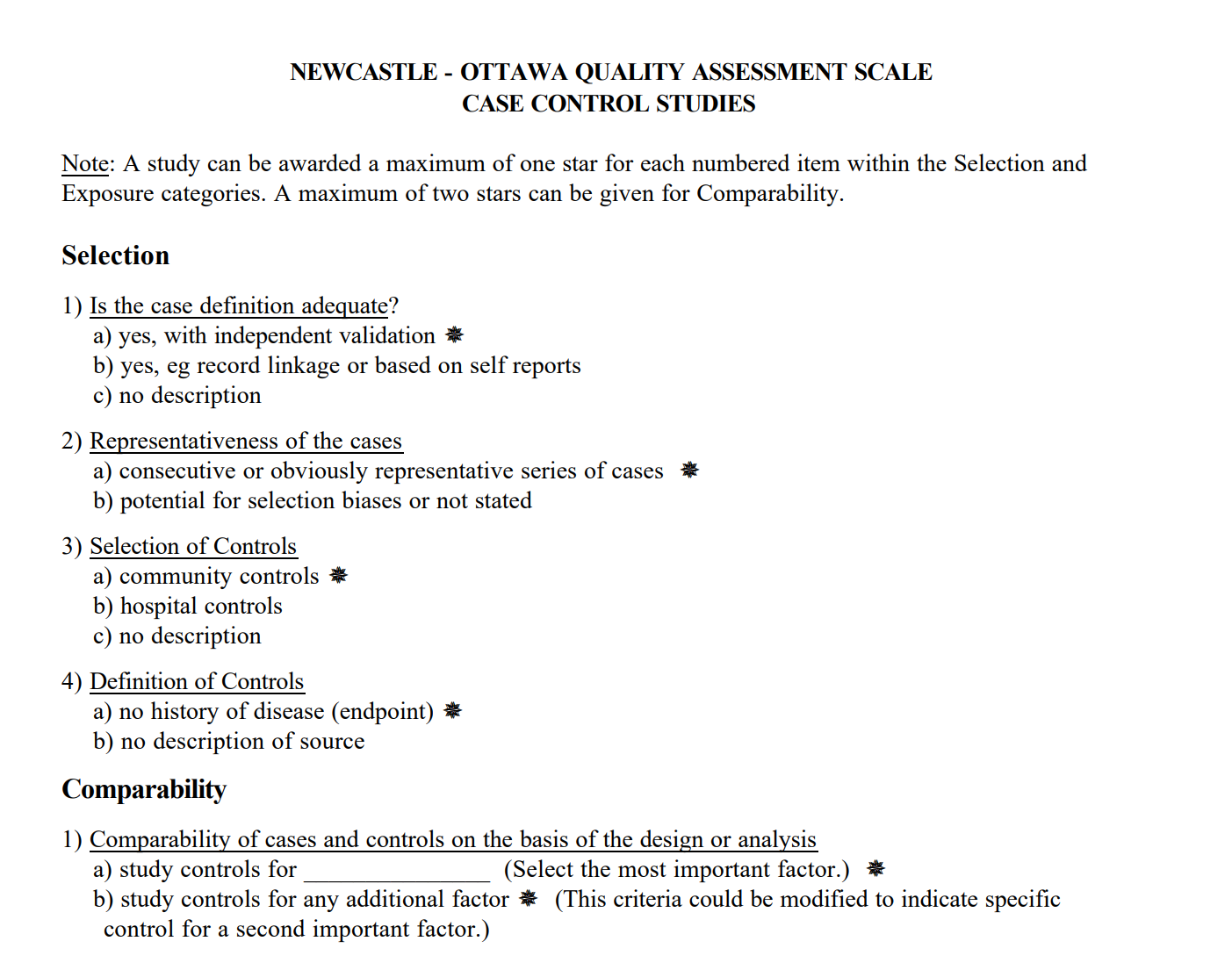


| **Author** | **GRADE quality score** | **1** | **2** | **3** | **4** | **Comparability 1 (a= reproductive pathology, b=age)** | **Total score**  **out of 6** | |
| --- | --- | --- | --- | --- | --- | --- | --- | --- |
| Aghajanova 1 | Low | a | b | a | a | a | 4 | Fair |
| Aghajanova 2 | Low | a | b | a | a | a | 4 | Fair |
| Ali | Low | a | b | a | a | ab | 5 | Good |
| Barry-Kinsella | Low | a | a | b | a | a | 4 | Fair |
| Ceydeli | Low | a | b | a | a | b | 4 | Fair |
| Dixit | Low | a | b | b | a | ab | 4 | Fair |
| Dorostghoal | Low | a | a | a | a | a | 5 | Good |
| Driessen | Low | b | b | b | a | x | 1 | Poor |
| Du | Low | a | b | a | a | ab | 5 | Good |
| El Mazny | Low | a | b | a | a | ab | 5 | Good |
| Feroze-Zaidi | Low | a | b | ab | a | x | 3 | Fair |
| Gimenes | Low | a | b | a | a | a | 4 | Fair |
| Graham | Low | a | b | a | a | a | 4 | Fair |
| Hambartsoumiam | Low | a | b | a | a | ab | 5 | Good |
| Hamilton | Low | a | b | a | a | ab | 5 | Good |
| Karaoglan | Low | a | b | b | b | x | 1 | Poor |
| Kilic | Low | a | b | a | a | a | 4 | Fair |
| Kralickova | Low | b | b | a | a | a | 3 | Fair |
| Kusuhara 1 | Low | b | b | a | a | x | 2 | Poor |
| Kusuhara 2 | Low | b | b | b | b | x | 0 | Poor |
| Laird | Low | a | b | b | a | b | 3 | Fair |
| Lessey | Low | a | b | b | a | x | 2 | Poor |
| Li 2 x | Low | a | b | a | a | x | 3 | Fair |
| Li 3 x | Low | a | a | a | a | ab | 6 | Good |
| Margioula-Siarkou | Low | a | a | b | a | ab | 5 | Good |
| Maynard | Low | a | b | a | a | x | 3 | Fair |
| Mikolajczyk | Low | a | b | b | a | a | 3 | Fair |
| Murto | Low | a | b | b | b | a | 2 | Poor |
| Petousis | Low | a | b | c | a | ab | 4 | Fair |
| Raine-Fenning | Low | a | b | a | a | a | 4 | Fair |
| Sahin | Low | a | b | b | a | ab | 4 | Fair |
| Steck | Low | a | b | a | a | a | 4 | Fair |
| Tawfeek | Low | a | b | a | a | a | 4 | Fair |
| Tsai | Low | b | a | a | a | ab | 5 | Good |
| Uysal | Low | a | a | a | a | ab | 6 | Good |
| Zebitay | Low | a | a | a | a | ab | 6 | Good |

National Heart, Lung and Blood Institute (NHLBI) Scoring for uncontrolled studies

Uncontrolled studies were assessed using the NHBLI ‘Quality Assessment Tool for Observational Cohort and Cross-sectional Studies’ criteria. There was a maximum score allowed of 14 points however 2 of these questions related to exposure and therefore were not relevant (highlighted), leading to a maximum score of 12. We categorised the studies as follows:

‘Good’ to 11 and 12 out of 12

‘Fair’ to 9 and 10 out of 12

‘Poor’ to 8 and below out of 12

NHLBI Scoring system:

1.Was the research question or objective in this paper clearly stated?

2. Was the study population clearly specified and defined?

3. Was the participation rate of eligible persons at least 50%?

4. Were all the subjects selected or recruited from the same or similar populations (including the same time period)? Were inclusion and exclusion criteria for being in the study prespecified and applied uniformly to all participants?

5. Was a sample size justification, power description, or variance and effect estimates provided?

6. For the analyses in this paper, were the exposure(s) of interest measured prior to the outcome(s) being measured?

7. Was the timeframe sufficient so that one could reasonably expect to see an association between exposure and outcome if it existed?

8. For exposures that can vary in amount or level, did the study examine different levels of the exposure as related to the outcome (e.g., categories of exposure, or exposure measured as continuous variable)?

9. Were the exposure measures (independent variables) clearly defined, valid, reliable, and implemented consistently across all study participants?

10. Was the exposure(s) assessed more than once over time?

11. Were the outcome measures (dependent variables) clearly defined, valid, reliable, and implemented consistently across all study participants?

**12. Were the outcome assessors blinded to the exposure status of participants? NA**

13. Was loss to follow-up after baseline 20% or less?

**14. Were key potential confounding variables measured and adjusted statistically for their impact on the relationship between exposure(s) and outcome(s)? NA**

| **Author** | **1** | **2** | **3** | **4** | **5** | **6** | **7** | **8** | **9** | **10** | **11** | **12** | **13** | **14** | **Total** | **Quality** |
| --- | --- | --- | --- | --- | --- | --- | --- | --- | --- | --- | --- | --- | --- | --- | --- | --- |
| Hirama | y | y | na | n/y | n | y | y | y | y | y | y | na | n | na | 10 | fair |
| Klentzeris | y | y | na | n/y | n | y | y | y | y | y | y | na | n | na | 10 | fair |
| Ordi | y | y | y | yy | n | y | y | y | y | y | y | na | n | na | 12 | good |
| Haxton | n | y | na | n/y | n | y | y | y | y | y | y | na | n | na | 9 | fair |
| Li 1 | y | y | y | yy | n | y | y | y | y | y | y | na | n | na | 12 | good |

Overall results:

‘Good’ – 11/38 studies ‘Fair’ – 22/38 studies ‘Poor’ - 5/38 studies

| **Author** | **GRADE quality score** |
| --- | --- |
| Hirama | Low |
| Klentzeris | Low |
| Ordi | Low |
| Haxton | Low |
| Li 1 | Low |
