# Supplementary material for: Endogenous progesterone in unexplained infertility: a systematic review and meta-analysis
Source: J Assist Reprod Genet. 2022 Dec 27;40(3):509–24. doi: 10.1007/s10815-022-02689-5 (PMC10033797; doi:10.1007/s10815-022-02689-5)
Supplement: Supplementary file 4 — List of included studies (DOCX 28 KB) [file 10815_2022_2689_MOESM4_ESM.docx]

| **Study ID** | **Study** | **Reference** |
| --- | --- | --- |
| 1 | Aghajanova 1 | Aghajanova L, Rumman A, Altmäe S, Wånggren K, Stavreus-Evers A. Diminished endometrial expression of ghrelin and ghrelin receptor contributes to infertility. Reproductive sciences (Thousand Oaks, Calif). 2010;17(9):823-32. |
| 2 | Aghajanova 2 | Aghajanova L, Altmäe S, Bjuresten K, Hovatta O, Landgren B-M, Stavreus-Evers A. Disturbances in the LIF pathway in the endometrium among women with unexplained infertility. Fertility and sterility. 2009;91(6):2602-10. |
| 3 | Ali | Ali R, Iqbal MUN, Rehman R, Khan TA. Association of novel stop-gained leukaemia inhibitory factor receptor gene (rs121912501) variant, leukaemia inhibitory factor and ovarian steroids with unexplained infertility among Pakistani women. Int J Clin Pract. 2021 Jul;75(7):e14245 |
| 4 | Barry-Kinsella | Barry-Kinsella C, Sharma SC, Cottell E, Harrison RF. Mid to late luteal phase steroids in minimal stage endometriosis and unexplained infertility. European journal of obstetrics, gynecology, and reproductive biology. 1994;54(2):113-8. |
| 5 | Ceydeli | Ceydeli N, Kaleli S, Calay Z, Erel CT, Akbas F, Ertungealp E. Difference in alpha(v)beta3 integrin expression in endometrial stromal cell in subgroups of women with unexplained infertility. European journal of obstetrics, gynecology, and reproductive biology. 2006;126(2):206-11. |
| 6 | Dhorostgoal | Dorostghoal M, Ghaffari HO, Marmazi F, Keikhah N. Overexpression of Endometrial Estrogen Receptor-Alpha in The Window of Implantation in Women with Unexplained Infertility. Int J Fertil Steril. 2018;12(1):37-42. |
| 7 | Dixit | Dixit SG, Ghatak S, Singh P, Bhattacharya S. Estrogen receptor, progesterone receptor and CD8+ expression in endometrium of women of unexplained infertility. Journal of gynecology obstetrics and human reproduction. 2018;47(10):533-7. |
| 8 | Driessen | Driessen F, Kremer J, Alsbach GP, de Kroon RA. Serum progesterone and oestradiol concentrations in women with unexplained infertility. British journal of obstetrics and gynaecology. 1980;87(7):619-23. |
| 9 | Du | Du JW, Tao XR, Xu KY, Fang LY, Qi XL. Polymorphisms in estrogen receptor-α are associated with idiopathic female infertility. Mol Med Rep. 2011;4(6):1239-42. |
| 10 | El Mazny | El-Mazny A, Abou-Salem N, Elshenoufy H. Doppler study of uterine hemodynamics in women with unexplained infertility. European journal of obstetrics, gynecology, and reproductive biology. 2013;171(1):84-7. |
| 11 | Feroze-Zaidi | Feroze-Zaidi F, Fusi L, Takano M, Higham J, Salker MS, Goto T, et al. Role and regulation of the serum- and glucocorticoid-regulated kinase 1 in fertile and infertile human endometrium. Endocrinology. 2007;148(10):5020-9. |
| 12 | Gimenes | Gimenes C, Bianco B, Mafra FA, Rosset V, Christofolini DM, Barbosa CP. The progins progesterone receptor gene polymorphism is not related to endometriosis-associated infertility or to idiopathic infertility. Clinics (Sao Paulo, Brazil). 2010;65(11):1073-6. |
| 13 | Graham | Graham RA, Seif MW, Aplin JD, Li TC, Cooke ID, Rogers AW, et al. An endometrial factor in unexplained infertility. BMJ (Clinical research ed). 1990;300(6737):1428-31. |
| 14 | Hambartsoumiam | Hambartsoumian E. Endometrial leukemia inhibitory factor (LIF) as a possible cause of unexplained infertility and multiple failures of implantation. American journal of reproductive immunology (New York, NY : 1989). 1998;39(2):137-43. |
| 15 | Hamilton | Hamilton MP, Fleming R, Coutts JR, Macnaughton MC, Whitfield CR. Luteal cysts and unexplained infertility: biochemical and ultrasonic evaluation. Fertility and sterility. 1990;54(1):32-7. |
| 16 | Haxton | Haxton MJ, Fleming R, Hamilton MP, Yates RW, Black WP, Coutts JR. Unexplained infertility' results of secondary investigations in 95 couples. British journal of obstetrics and gynaecology. 1987;94(6):539-42. |
| 17 | Hirama | Hirama Y, Ochiai K. Estrogen and progesterone receptors of the out-of-phase endometrium in female infertile patients. Fertility and sterility. 1995;63(5):984-8. |
| 18 | Karaoglan | Karaoğlan Ö, Kuyucu Y, Ürünsak İF, Gümürdülü D, Tap Ö. Morphological features of the secretory phase endometrium in women with unexplained infertility. Ultrastruct Pathol. 2021 May 4;45(3):243-256. |
| 19 | Kilic | Kilic S, Hatipoglu T, Erdogan D, Elmas C, Yuksel B, Tasdemir N, et al. Impact of high levels of progesterone on alpha(1)-integrin distribution in the endometrium of patients with unexplained infertility. Acta histochemica. 2008;110(5):363-70. |
| 20 | Klentzeris | Klentzeris LD, Bulmer JN, Seppälä M, Li TC, Warren MA, Cooke ID. Placental protein 14 in cycles with normal and retarded endometrial differentiation. Human reproduction (Oxford, England). 1994;9(3):394-8. |
| 21 | Kralickova | Kralickova M, Sima R, Vanecek T, Sima P, Rokyta Z, Ulcova-Gallova Z, et al. Leukemia inhibitory factor gene mutations in the population of infertile women are not restricted to nulligravid patients. European journal of obstetrics, gynecology, and reproductive biology. 2006;127(2):231-5. |
| 22 | Kusuhara 1 | Kusuhara K. Luteal function in infertile patients with endometriosis. American journal of obstetrics and gynecology. 1992;167(1):274-7. |
| 23 | Kusuhara 2 | Kusuhara K. Clinical importance of endometrial histology and progesterone level assessment in luteal-phase defect. Hormone research. 1992;37. |
| 24 | Laird | Laird SM, Tuckerman EM, Dalton CF, Dunphy BC, Li TC, Zhang X. The production of leukaemia inhibitory factor by human endometrium: presence in uterine flushings and production by cells in culture. Human reproduction (Oxford, England). 1997;12(3):569-74. |
| 25 | Lessey | Lessey BA, Castelbaum AJ, Sawin SW, Sun J. Integrins as markers of uterine receptivity in women with primary unexplained infertility. Fertility and sterility. 1995;63(3):535-42. |
| 26 | Li 1 | Li TC, Lenton EA, Dockery P, Cooke ID. A comparison of some clinical and endocrinological features between cycles with normal and defective luteal phases in women with unexplained infertility. Human reproduction (Oxford, England). 1990;5(7):805-10. |
| 27 | Li 2 | Li TC, Dockery P, Cooke ID. Endometrial development in the luteal phase of women with various types of infertility: comparison with women of normal fertility. Human reproduction (Oxford, England). 1991;6(3):325-30. |
| 28 | Li 3 | Li TC, Lenton EA, Dockery P, Rogers AW, Cooke ID. The relation between daily salivary progesterone profile and endometrial development in the luteal phase of fertile and infertile women. British journal of obstetrics and gynaecology. 1989;96(4):445-53. |
| 29 | Margioula-Siarkou | Margioula-Siarkou C, Prapas Y, Petousis S, Milias S, Ravanos K, Dagklis T, Kalogiannidis I, Mavromatidis G, Haitoglou C, Prapas N, Rousso D. LIF endometrial expression is impaired in women with unexplained infertility while LIF-R expression in all infertility sub-groups. Cytokine. 2017 Aug;96:166-172. |
| 30 | Maynard | Maynard PV, Baker PN, Symonds EM, Sant-Cassia LJ, Johnson J, Selby C. Nuclear progesterone uptake by endometrial tissue in cases of subfertility. Lancet (London, England). 1983;2(8345):310-2. |
| 31 | Mikolajczyk | Mikołajczyk M, Skrzypczak J, Szymanowski K, Wirstlein P. The assessment of LIF in uterine flushing--a possible new diagnostic tool in states of impaired fertility. Reproductive biology. 2003;3(3):259-70. |
| 32 | Murto | Murto T, Bjuresten K, Landgren B-M, Stavreus-Evers A. Predictive value of hormonal parameters for live birth in women with unexplained infertility and male infertility. Reproductive biology and endocrinology : RB&E. 2013;11:61. |
| 33 | Ordi | Ordi J, Creus M, Ferrer B, Fábregues F, Carmona F, Casamitjana R, et al. Midluteal endometrial biopsy and alphavbeta3 integrin expression in the evaluation of the endometrium in infertility: implications for fecundity. Int J Gynecol Pathol. 2002;21(3):231-8. |
| 34 | Petousis | Petousis S, Prapas Y, Margioula-Siarkou C, Ravanos K, Milias S, Mavromatidis G, et al. Unexplained infertility patients present the mostly impaired levels of progesterone receptors: Prospective observational study. Am J Reprod Immunol. 2018;79(6):e12828. |
| 35 | Raine-Fenning | Raine-Fenning NJ, Campbell BK, Kendall NR, Clewes JS, Johnson IR. Endometrial and subendometrial perfusion are impaired in women with unexplained subfertility. Human reproduction (Oxford, England). 2004;19(11):2605-14. |
| 36 | Sahin | Sahin ME, Madendag IC, Sahin E, Madendag Y, Karakukcu C. The role of serum progesterone induced blocking factor on unexplained infertility. European Journal of Obstetrics and Gynecology and Reproductive Biology. 2020;252:15-8. |
| 37 | Steck | Steck T, Giess R, Suetterlin MW, Bolland M, Wiest S, Poehls UG, et al. Leukaemia inhibitory factor (LIF) gene mutations in women with unexplained infertility and recurrent failure of implantation after IVF and embryo transfer. European journal of obstetrics, gynecology, and reproductive biology. 2004;112(1):69-73. |
| 38 | Tawfeek | Tawfeek MA, Eid MA, Hasan AM, Mostafa M, El-Serogy HA. Assessment of leukemia inhibitory factor and glycoprotein 130 expression in endometrium and uterine flushing: a possible diagnostic tool for impaired fertility. BMC women's health. 2012;12:10. |
| 39 | Tsai | Tsai HD, Chang CC, Hsieh YY, Lo HY. Leukemia inhibitory factor expression in different endometrial locations between fertile and infertile women throughout different menstrual phases. Journal of assisted reproduction and genetics. 2000;17(8):415-8. |
| 40 | Uysal | Uysal S, Ozbay EPÖ, Ekinci T, Aksüt H, Karasu S, Işık AZ, et al. Endometrial spiral artery Doppler parameters in unexplained infertility patients: is endometrial perfusion an important factor in the etiopathogenesis? Journal of the Turkish German Gynecological Association. 2012;13(3):169 |
| 41 | Zebitay | Zebitay AG, Tutumlu M, Verit FF, Ilhan GK, Gungor ES, Cetin O, et al. A comparative analysis of arterial blood flow in unexplained infertility, tubal infertility and fertile groups. Gynecological endocrinology : the official journal of the International Society of Gynecological Endocrinology. 2016;32(6):442-5. |
