# Supplementary material for: Endogenous progesterone in unexplained infertility: a systematic review and meta-analysis
Source: J Assist Reprod Genet. 2022 Dec 27;40(3):509–24. doi: 10.1007/s10815-022-02689-5 (PMC10033797; doi:10.1007/s10815-022-02689-5)
Supplement: Supplementary file 5 — Prisma Flowchart (DOC 58 KB) [file 10815_2022_2689_MOESM5_ESM.doc]

**
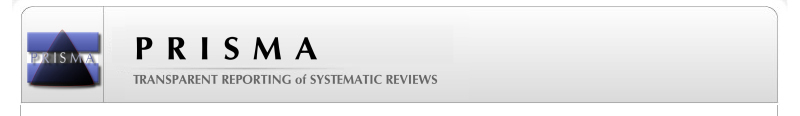
What is the role of Progesterone in Unexplained Infertility?**

**Screening**

**Included**

**Eligibility**

**Identification**

Records screened
(n = 526)

Full-text articles excluded, with reasons
(n = 78)

15 – no full paper

49 – not relevant or results for UI not analysed separately

10 – not in English

4 – no access to full paper

Studies included in qualitative synthesis
(n = 41)

Records identified through database searching
(n = 493)

Additional records identified through other sources
(n = 33)

Records excluded
(n = 407)

Full-text articles assessed for eligibility
(n = 119)
